# The complete mitochondrial genome of long-tailed red-toothed shrew (*Episoriculus leucops)* and implication of phylogenetic status

**DOI:** 10.1080/23802359.2021.1907798

**Published:** 2021-03-31

**Authors:** Dan Chen, Boxin Qin, Fei Xie, Qiong Wang, Shunde Chen

**Affiliations:** College of Life Sciences, Sichuan Normal University, Chengdu, China

**Keywords:** Bayesian phylogenetic tree, phylogenetic analysis, evolution

## Abstract

We determined a complete mitochondrial genome of *Episoriculus leucops*. This mitogenome is a circular molecule with 16,838 bp in length, containing 13 protein-coding genes, 22 transfer RNA genes (tRNA), and two ribosomal RNA genes (rRNA). This mitochondrial genome has a base composition of 32.8% A, 29.2% T, 24.9% C, and 13.1% G. We reconstructed Bayesian phylogenetic tree by taking advantage of 19 species belonging to subfamily Soricinae. Phylogenetic tree shows that the long-tailed red-toothed shrew belongs to genus *Episoriculus*, and it is the closest relationship with *E. caudatus.* This mitochondrial genome provides an important resource for addressing taxonomic issues and studying molecular evolution.

The long-tailed red-toothed shrew, *Episoriculus leucops* (Horsfield 1855), belongs to Soricinae. The species has been recorded from Tibet to Sichuan and Yunnan (Wang 2003; Smith and Xie 2009). Its type Locality is in Nepal (Hoffmann 1985). In China, the species can be found in moist coniferous forests, azalea forests as well as broad-leaved deciduous forests at elevations ranging from 3000 to 3500 m. The species also lives in moist dwarf bamboo forests, shrubs and grasslands, and it is said that the species also inhabit rural and cultivated areas (Smith and Xie 2009).

An individual was caught in a barrack of Lushui County, Nujiang Lisu Autonomous Prefecture in Yunnan Province with an altitude of 2250 m (Latitude: 25.95919°N, Longitude: 98.71446°E). The specimen of *E. leucops* was deposited at Sichuan Academy of Forestry, Chengdu (Shaoying Liu, shaoyliu@163.com) under the voucher number SAF18058. The DNA of *E. leucops* was deposited at Sichuan Normal University, Chengdu (Shunde Chen, csd111@126.com) under the voucher number csd2365.

This mitochondrial genome provides an important resource for addressing taxonomic issues and studying molecular evolution. The total DNA of *E. leucops* was extracted by TRIzol® Reagent. The Illumina Hiseq 4000 sequencing platform was used for mitotic genome sequencing. The SPAdes V3.10.1 (Nurk et al. 2013) and The GapCloser V1.12 (Luo et al. 2012) were used for genome assembly. The Illumina TruSeq™ Nano DNA Sample Prep Kit was used for library construction.The amino acid sequence of the sample is compared with known protein databases (e.g. NR, Swiss-Prot, eggNOG, KEGG, GO databases) for blastp (evalue < = 1e-5), and the functional annotation information of the coding gene with higher reliability is obtained. The whole mitogenome of *E. leucops* is 16,838 base pairs (bp), including 13 protein-coding genes, two ribosomal RNA genes, and 22 transfer RNA genes. Its base composition is 32.8% A, 29.2% T, 24.8% C, and 13.1% G. The 13 protein-coding genes is 11,349 bp, and all of these protein-coding genes start translation with ATG, except for ND5 (ATA) and ND3 (ATT). Three types of termination codons were used, including TAA for COX1, COX2, ATP8, ND4L, ND3, ND5, and ND6; CAA for ATP8, and ND2; AGA for Cyt b. The two non-coding regions are comprised of a light strand replication origin (OL) and a D-loop region. The light strand replication origin (OL), 40 bp in length, is located between tRNA^Asn^ and tRNA^Cys^. The D-loop region(1367 bp), is located between tRNA^Pro^ and tRNA^Phe^.

Thirteen mitochondrial protein genes from *E. leucops* and other 22 species were used for phylogenetic reconstruction. Three species of genus *Crodura* were taken as outgroups, namely *C. sibirica, C. tanakae* and *C. fuliginosa*. The jModeltest 2.1.3 (Darriba et al. 2012) was used to determine the model of evolution by Akaike Information Criterion (AIC). The best substitution model was GTR. BEAST v1.6.1 (Drummond et al. 2012) was applied to the reconstructed Bayesian phylogenetic tree. The Bayesian phylogenetic tree shows that *E. leucops* belongs to subfamily Soricinae, and *E. leucops* is sister to *E. caudatus* with well supported value (BPP = 1.00) (Figure 1).

**Figure 1.**
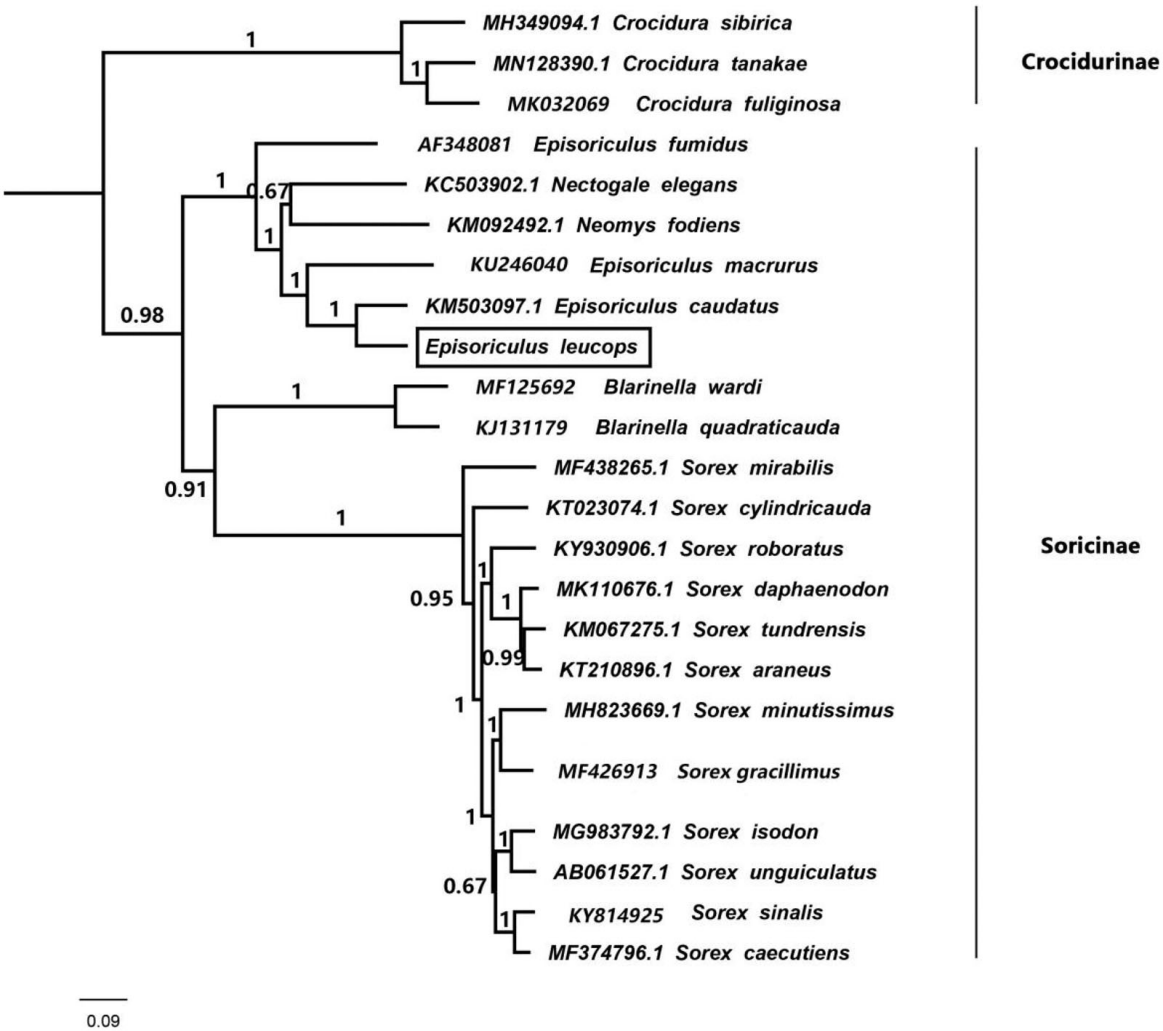
Bayesian phylogenetic tree based on 22 protein genes of mitochondrial genome. Numbers by the nodes indicate Bayesian posterior probabilities.

## Data Availability

The genome sequence data that support the findings of this study are openly available in GenBank of NCBI at (https://www.ncbi.nlm.nih.gov/) under the accession no. MW114663. The associated BioProject, SRA, and Bio-Sample numbers are PRJNA698717, SUB9018911, and SAMN17734837, respectively.
